# Evaluation of the Safety and Impact of Heat-Treated *Lactiplantibacillus plantarum* KM2 Fermentation on Gut Microbiome Architecture

**DOI:** 10.4014/jmb.2411.11069

**Published:** 2024-12-27

**Authors:** Seok Won, Yejin Jeong, Ji-Eun Kim, Jong-Hoon Kim, Hyun Soo Song, Hyun-Hwa Bae, Mi-Sun Kwak, Don-Kyu Kim, Moon-Hee Sung, Suryang Kwak

**Affiliations:** 1 Department of Bio and Fermentation Convergence Technology, College of Science and Technology, Kookmin University, Seoul 02707, Republic of Korea; 2 Microbiome Technology Research Institute, Kookmin Bio, Seoul 02826, Republic of Korea; 3 Department of Physical Medicine and Rehabilitation, Chung-Ang University Gwangmyeong Hospital, Gwangmyeong 14353, Republic of Korea

**Keywords:** Postbiotics, *Lactiplantibacillus plantarum*, safety, gut microbiome

## Abstract

Postbiotics, bioactive compounds from the fermentation process by probiotics, are gaining attention for their potential health benefits as safer alternatives to live probiotic microbes. *Lactiplantibacillus plantarum* is a well-studied probiotic species known for promoting gut health and immune modulation. However, the safety and effects of its postbiotic formulations on the gut microbiome structure remain less explored. This study presents a randomized, double-blind, placebo-controlled human study of KLP-KM2, a postbiotic consisting of heat-treated *L. plantarum* KM2 fermentation complex, in elderly participants. Over 12 weeks, KLP-KM2 consumption did not result in noticeable adverse reaction cases compared to the placebo. Nevertheless, the gut microbial diversity and taxonomic architecture of the KLP-KM2 recipients were differentiated from those of the placebo recipients after 12 weeks. A notable outcome was the increase in the number of subjects carrying *Veillonella* spp., which contributed to the distinct gut microbiome profiles observed between the two groups. Interestingly, KLP-KM2 facilitated the de novo colonization of *Veillonella* spp. in subjects who had not harbored these bacteria at the baseline. These results suggest the potential of KLP-KM2 as a safe and effective postbiotic intervention to enhance energy metabolism and mobility in older adults.

## Introduction

*Lactiplantibacillus plantarum* is a probiotic lactic acid bacterium found in various fermented foods and the human gastrointestinal tract and widely recognized for its probiotic properties and health-promoting benefits, such as supporting microbiome balance, gut barrier function, and immune modulation [1-6]. This species is also noted for its resilience in harsh gastrointestinal conditions, allowing it to survive gastric acid and bile salts, which are key features of effective probiotics . In addition, multiple studies highlighted *L. plantarum*’s ability to produce bioactive compounds which contribute to its capacity to inhibit pathogenic bacteria and promote mucosal health [7-11]. These qualities make *L. plantarum* a promising probiotic in functional foods and therapeutic products [12]. The safety profile of *L. plantarum* is generally favorable, as its non-pathogenic nature minimizes infection risks in healthy populations [13]. However, multiple clinical cases have demonstrated that in some certain individuals with weakened immune systems may face a low but tangible risk of bacteremia if live *L. plantarum* translocate across the gut barrier [14-16].

Postbiotic formulation of *L. plantarum* can serve as a safer alternative to avoid the potential risks associated with its regimen as a probiotic. Unlike live probiotics, postbiotics—non-viable bacterial components like cell wall fragments, metabolites, or lysates—reduce the risk of infection or undesired microbial translocation, which can occasionally be a concern with probiotics especially in vulnerable populations with weakened immune systems [17, 18]. In addition, postbiotic formulations are also more stable, with a longer shelf life, consistent efficacy, and lower contamination risks, as they don’t require preservation of live cultures they do not necessitate the preservation of viable cultures [19, 20]. Still, it is crucial to carefully assess the safety of postbiotics, as bioactive metabolites in postbiotic formulations could theoretically cause allergic reactions, gastrointestinal discomfort, or immune responses, particularly at high doses [21]. While multiple studies have demonstrated the safety of *L. plantarum* postbiotics in various animal models, their safety in humans has yet to be evaluated [22–26].

The current study aims to investigate the safety and effects of KLP-KM2, the postbiotic formulation of *L. plantarum* KM2 isolated from a low-temperature-aged beef [27, 28], through a placebo-controlled human study in adults aged 50 and above. KM2 is a *L. plantarum* strain isolated from a low-temperature aging beef, and its genetic and physiological characteristics were recently uncovered [27, 28]. Another recent murine model study found that KLP-KM2 administration significantly mitigates dexamethasone-induced muscle atrophy in mice by enhancing muscle mass, strength, and gut microbiota diversity [29]. Through the human study, we demonstrated the safety of KLM-KM2 in human and its impact on gut microbiome structure, offering insights into potential mechanisms underlying its benefits. This study is the first to validate the safety of *L. plantarum*-based postbiotics in humans.

## Materials and Methods

### Production of KLP-KM2

**Fig. 1:**
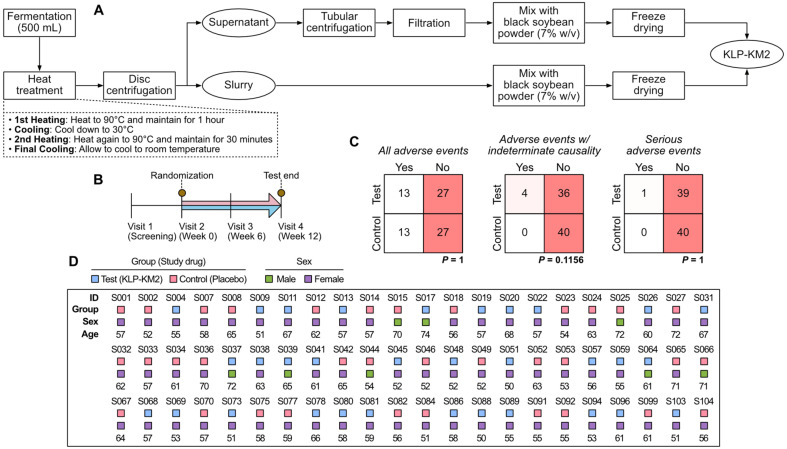
Study design for evaluation of the safety of KLP-KM2 and its impact on gut microbiome. (**A**) Scheme of the KLP-KM2 preparation process. (**B**) Timeline of the KLP-KM2 human study. Subjects were recruited and screened at the first visit, randomly assigned for the test group (KLP-KM2 administration) or control group (placebo administration) at the second visit. Their fecal specimens were collected 2 times (brown circles). (**C**) Statistical comparisons about the occurrence of adverse events for 12 weeks between the two groups. Chi-Squared test (the case of all adverse events) and Fisher’s exact test (other two comparison cases) were used complementarily depending on the size of the expected frequencies. (**D**) Visual description of the per protocol set of this study.

KLP-KM2 and placebo was produced by Kookmin Bio GMP Factory (Republic of Korea). The production process of KLP-KM2 was described in detail in Fig. 1A. Black soybean powder (7% w/v) was utilized as a formulation excipient during the preparation of study drugs (Fig. 1A), considering its human safety profile and suitability for incorporating the postbiotic substances from *L. plantarum* KM2 fermentation. Chemical compositions of KLP-KM2 and placebo were described in Table 1.

### Study Cohorts and Fecal Samples

This human study was approved by the Korea Disease Control and Prevention Agency, and registered in Clinical Research Information Service (KCT0009906). Chung-Ang University Gwangmyeong Hospital approved the study protocol named KLBiome_KLP-KM2 for this human study. A total of 80 participants who met the inclusion criteria were recruited and allocated to either test or control group at visit 2 (week 0), based on the assignment code generated through the block randomization method. To ensure balanced randomization between the groups, the participant numbers in each group were kept equal at a 1:1 ratio. The randomization table was created in advance using the SAS system’s randomization program. Participants consumed a total of 9 g of either KLP-KM2 (test group) or placebo (control group), divided into two doses per day, according to the assigned group (Fig. 1A and 1B). Further details about the randomized, double-blind, placebo-controlled design of the current human study were described in Supplementary Information.

### Evaluation of the Adverse Event Profile of KLP-KM2 in Human Subjects

All treatment emergent adverse events that occurred after the study drug administration were coded based on MedDRA v27.0. Adverse events were monitored through non-directive questioning at each visit during the human study. Adverse events were identified via self-reports from participants, physical examinations, clinical pathology assessments, and other diagnostic evaluations conducted both during and between visits. Recorded details included information on the onset and resolution dates, severity, outcome, any interventions related to study drugs, causality assessment between the events and study drugs, suspected medications other than study drugs, and any treatments given, including the type and specifics of such treatment. In addition, clinical pathology tests for safety assessment, such as hematological tests, biochemical tests, and urinalysis, were conducted at visits 1 and 4.

### Shotgun Metagenomic Sequencing and Data Analyses

Fecal samples were collected, and their metagenomic DNAs were extracted, subjected to shotgun sequencing, and preprocessed by Sanigen (Republic of Korea). Specifically, the metagenomic DNAs were extracted from fecal samples using the TruSeq Nano DNA prep kit of Illumina. The library preparation stage involves an adapter ligation step with 8 cycles of PCR amplification. The constructed libraries were then checked for size using the Tapestation 4200 instrument, and those with a length of 450-650 bp were selected for sequencing. The filtered libraries were pooled and analyzed via 2 × 150 bp paired-end sequencing on an Illumina NextSeq 2000. The raw reads were processed using Trimmomatic v0.39 [30] to remove low-quality reads and adapter sequences. To remove the PhiX sequences added during the sequencing process, the trimmed reads were aligned to the PhiX reference genome (NC_001422.1) using BWA v0.7.17 (r1188) [31], and the aligned reads were removed using SAMtools v1.15.1 [32]. Taxonomic classification is performed using Kraken2 v2.1.2 [33], and the species abundance is estimated using Bracken v2.55 [34].

Microbiome alpha and beta diversities were computed using the vegan [35] and ape [36] packages, and analysis results were visualized using ggplot2 [37] in R 4.1.0. Permutational multivariate analysis of variance (PERMANOVA) was conducted using adonis2 in the vegan [35] for group comparisons in principal coordinates analyses (PCoAs) with the Bray-Curtis index. MaAsLin2 (Multivariate Association with Linear Models) [38] was used to identify associations between metadata variables and microbial relative abundances.

## Results

### Collection of Study Cohorts and Their Fecal Metagenomic Sequencing Data

In the initial cohort of 80 subjects, 40 were randomly assigned to the control group and 40 to the test group. Metagenomes were extracted from fecal specimens of the subjects and analyzed via shotgun metagenomic sequencing. The depth of the demultiplexed, trimmed, and filtered sequences was ranged from 10,461,307 to 22,095,686 reads (median 14,723,473 reads). Rarefaction analysis based on the Shannon index after taxonomic classification via Bracken confirmed adequate sequencing read depths (Fig. S1).

### Placebo-Controlled Study Demonstrated the Safety of KLP-KM2 in the Elderly Cohort

The safety of KLP-KM2 was evaluated in all 80 subjects who consumed one of the study drugs at least once, and their adverse event rates were calculated. There was no association between the adverse event frequency and KLP-KM2; 13 subjects (32.5%) in both test and control groups reported adverse events (Fig. 1C). In the test group, adverse events in 9 out of 13 subjects were deemed clearly unrelated to KLP-KM2 administration, while a potential link could not be ruled out in 4 subjects (3 with indigestion and 1 with loose stools). In the control group, all 13 adverse reaction cases were unrelated to KLP-KM2 administration. A serious adverse event, a one-day hospitalization for a urinary tract infection was reported in the test group. However, it was determined by clinical investigators to be unrelated to KLP-KM2. Statistical analyses with 95% confidence demonstrated no association between the frequency of adverse reactions and KLP-KM2 at any level (Chi-Squared test and Fisher’s exact, Fig. 1C).

After 12 weeks of study drug administration, the test group showed 0.17 ± 1.76% increase in monocyte levels, while the control group decreased by 0.64 ± 1.74%, resulting in a statistically significant difference between the two groups (*P* = 0.0298, Wilcoxon Rank-Sum test). However, no subjects were deemed to have clinical significance by the medical professional. In addition, no statistically significant differences were observed between the groups in any of the other hematological, biochemical, or urinalysis test items. There were also no statistically considerable distinctions between the groups in vital signs (blood pressure and pulse rate) or body weight after 12 weeks, and both groups showed normal results in electrocardiogram tests after 12 weeks. Accordingly, KLP-KM2 administration was concluded to be safe for human use.

### KLP-KM2 Administration Resulted in an Altered Gut Microbiome Architecture

**Fig. 2:**
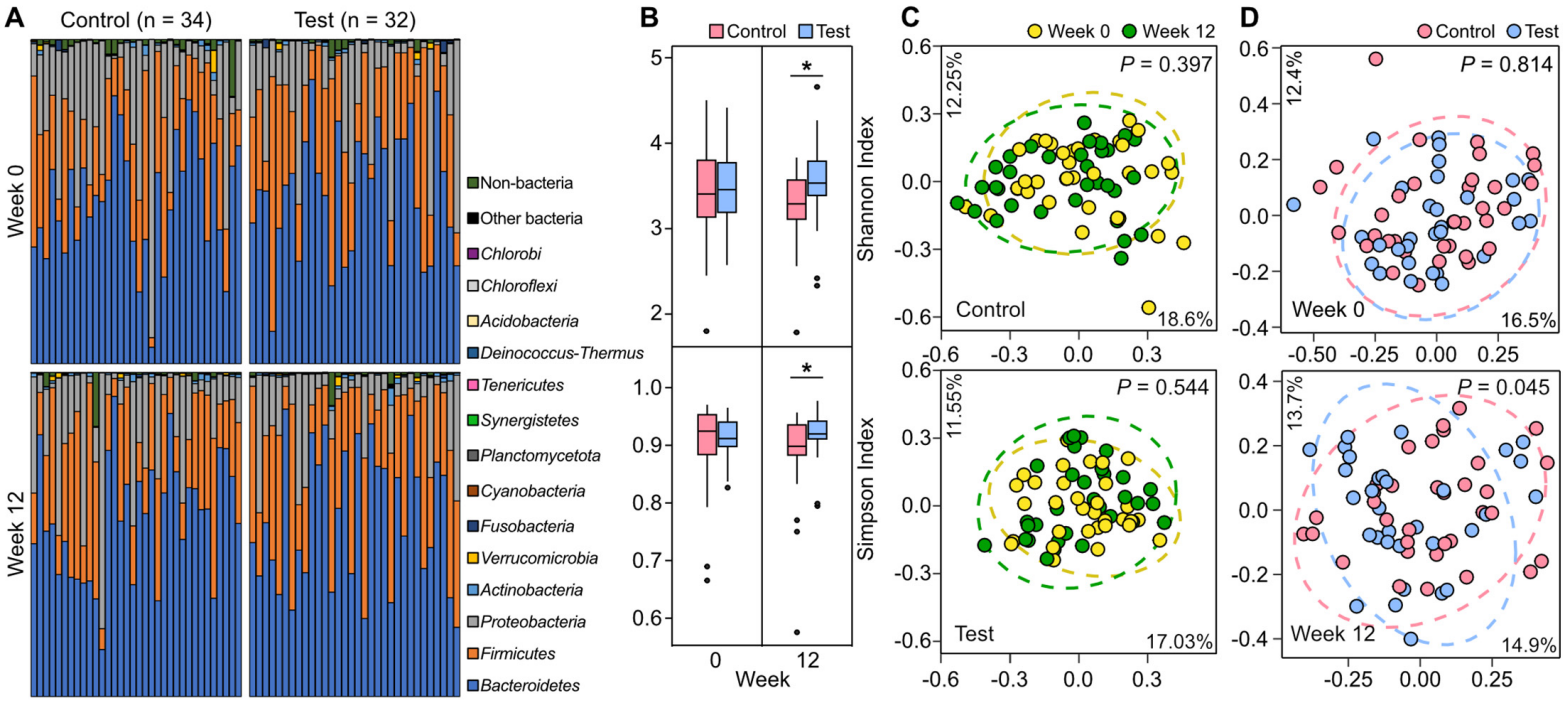
Comparison of the gut microbiome structures across subjects. (**A**) Bar charts of taxonomic compositions of control and test group subjects at the phylum level. (**B**) Alpha diversity levels of the cohort were compared between test and control groups at baseline (0 week), and end-point (12 week) of the clinical trial via Shannon and Simpson indices. An asterisk denotes a statistical significance between the groups (*p < 0.05, Wilcoxon Rank-Sum test). (**C** and **D**) Principal coordinate analysis for comparing gut microbiome taxonomic structures of subjects at week 0 and week 12 (**C**) and between the test and control groups (**D**) based on Bray-Curtis dissimilarity at the species level. Permutational analysis of variance revealed that gut microbiomes of both test and control groups did not structurally shift from baseline to a subsequent time point (alpha = 0.05).

A part of subjects was excluded due to reasons such as withdrawal of consent, low compliance (less than 70%), administration of antibiotics or prohibited concomitant medications exceeding the specified criteria during the human study. As a result, the per protocol (PP) set was established with 66 subjects with 32 in the test group and 34 in the control group. Metadata about the subjects in the PP set were described in Fig. 1D. At baseline, the gut microbiome taxonomic composition at the phylum level varied across all subjects in the PP set, but no prominent distinctions were observed between the control and test groups. *Firmicutes*, *Bacteroidetes*, and *Proteobacteria* account for major phylum compositions in the study cohort, irrespective of group assignment (Fig. 2A). Similarly, baseline alpha diversity levels and taxonomic architectures of the gut microbiomes were not statistically discernible between the two groups (Fig. 2B and 2C). These results of the diversity analyses confirm that the random allocation of subjects in this cohort study was not biased regarding the structural characteristics of the gut microbiome. On the other side, the test group exhibited higher alpha diversity levels compared to those of the control group after study drug administration for 12 weeks in the views of both Shannon (*P* = 0.016) and Simpson indices (*P* = 0.025, Fig. 2B). We compared gut microbiome architectures of baseline and week 12 in the two groups and found that both placebo and KLP-KM2 did not significantly alter gut microbiome architecture of their recipients for 12 weeks (Fig. 2C). However, it was observed that structural differences between the gut microbiomes of the two groups became statistically more significant during the 12 weeks administration of study drugs (Fig. 2D). These results suggest that the directionalities of structural shifts in the gut microbiome differed between the control and test groups, although both study drugs did not noticeably alter the recipients’ gut microbiomes for the 12 weeks.

### KLP-KM2 Administration Promoted the Acquisition of *Veillonella* spp.

**Fig. 3:**
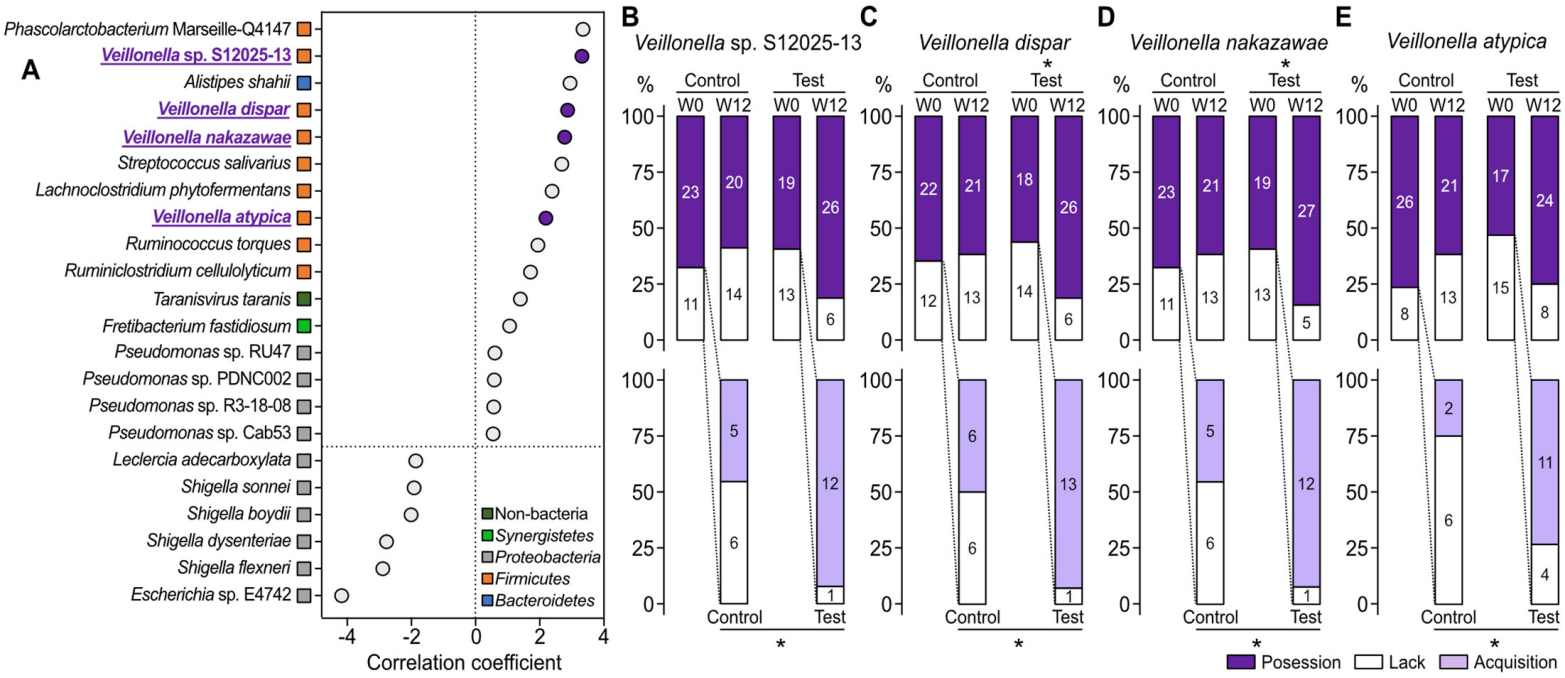
Screening and tracking taxonomic features associated with the KLP-KM2 administration. (**A**) Species whose changes in the relative abundance were associated with KLP-KM2 administration were screened via multivariable association with linear models (MaAsLin2, *p* < 0.05). Changes in relative abundances of multiple *Veillonella* species (highlighted in purple) were positively associated with the KLP-KM2 administration. (**B–E**) Presence or absence of the screened *Veillonella* species were tracked over the course of 12 weeks. *p < 0.05, Chi-squared test and Fiser’s exact test.

Next, we delved into determinants of the divergent shifts in gut microbiome architectures between the control and test groups by identifying taxa with relative abundance changes significantly correlated with KLP-KM2 administration, at the species level via general linear models [38]. Most species screened as features whose relative abundance changes were positively correlated with KLP-KM2 administration were *Firmicutes* in the phylum level (Fig. 3A). Intriguingly, among the screened *Firmicutes* taxonomic features, 4 *Veillonella* species which are known for their role in the lactate fermentation generating short-chain fatty acids (SCFAs) like acetate and propionate [39-42], were identified (Fig. 3A). *Veillonella* is a representative genus which can utilize lactate from the carbohydrate fermentation by other bacteria, although it does not metabolize carbohydrates directly [40, 42]. It implies that lactate in KLP-KM2, which is a postbiotic derived from the lactic acid bacteria fermentation, may be a factor contributing to the increase in the identified *Veillonella* taxa. We further investigated the potential relation of the identified *Veillonella* species to KLP-KM2 administration by tracking their absence and presence in the gut microbiomes of subjects. In the case of the subjects who did not harbor the 4 *Veillonella* species at baseline, KLP-KM2 administration significantly facilitated the subjects to newly acquire the species, compared to placebo (lower panels of Fig. 3B–3E). The acquisition led to an increased proportion of subjects carrying the corresponding *Veillonella* species exclusively in the test group, after consuming KLP-KM2 for 12 weeks. Specifically, the changes in the proportion of individuals harboring *V. dispar* and *V. nakazawae* were statistically significant in the test group only (upper panels of Fig. 3C and 3D). On the other hand, KLP-KM2 administration was associated with a reduced abundance of several *Shigella* species, namely *S. sonnei*, *S. boydii*, *S. dysenteriae*, and *S. flexneri* (Fig. 3A).

## Discussion

We investigated the impact of KLP-KM2, a postbiotic formulation of *L. plantarum* KM2, on the gut microbiome as well as its safety via a placebo-controlled human study. KLP-KM2 administration led to unique structural shift of gut microbiomes, with *Veillonella* species representing half of the top 8 taxa whose changes in relative abundance were positively correlated with KLP-KM2 administration. This phenomenon may stem from the postbiotic composition containing lactate, given the lactate-utilizing capacity of the *Veillonella* [43]; the identified *Veillonella* species are known for their role in the fermentation of lactate, which they convert into short-chain fatty acids (SCFAs), such as acetate and propionate [39-44]. This metabolic activity plays a critical role in the human physiology by maintaining gut health. Notably, *Veillonella atypica*, one of the most extensively studied species within the identified *Veillonella* species, is frequently detected in the gut microbiota of athletes [40, 41]. Its ability to metabolize lactate has been associated with improved exercise performance, potentially contributing to a delay in muscle fatigue during intense physical activity as well as the supply of energy source, namely SCFAs [41].

*Phascolarctobacterium* Marseille-Q4147, *Alistipes shahii*, *Streptococcus salivarius*, *Lachnoclostridium phxtofermentans*, *Ruminococcus torques*, and *Ruminiclostridium cellulolyticum* were taxonomic features exhibited correlation levels comparable to those observed in the *Veillonella* species, although their capability to utilize lactate has not been elucidated yet. Excepting *S. salivarius*, other 5 taxonomic features have also been well documented as SCFA producers or are part of genera known for their high SCFA production capacity [45-48]. Specifically, *A. shahii* predominantly produces succinate, with acetate and propionate as minor products [47], while acetate is the major metabolic end product in the genera *Lachnoclostridium*, *Ruminococcus*, and *Ruminiclostridium* [45, 46]. The genus *Phascolarctobacterium* showed a significant association with propionate concentration in fecal samples from male primates [48].

Although lactic acid bacteria, including *L. plantarum*, are widely used in various probiotic products based on the general perception of its safety, multiple clinical cases have described that it still poses potential risks, such as bacteremia, particularly for individuals with weakened immune systems [49, 50]. In older adults, immune function declines, due to immunosenescence, a gradual weakening of innate and adaptive responses, making them more susceptible to infections and slower to recover [51-53]. In this context, while probiotics are generally safe for older adults, caution may be necessary for individuals with frailty or those with certain underlying health conditions when considering live bacterial supplements [54-56]. Since postbiotics lack live organisms, they do not carry the potential risk of sepsis, bacteremia, or other infections that can occur with live probiotics, especially in at-risk populations including the elderly [50]. In the current placebo-controlled study, we statistically corroborated the safety of KLP-KM2, a postbiotic formulation of *L. plantarum* KM2, through a placebo-controlled human study involving a cohort including individuals in their 50s to 70s. These findings demonstrate concordance with the previous investigation of KLP-KM2 conducted through both in vitro and murine models [29]. Specifically, Jeong *et al*. reported enhanced gastrointestinal microbiota diversity following KLP-KM2 administration, as quantified by Shannon index calculations—a finding that parallels our current human study outcomes. Their investigation further elucidated KLP-KM2's therapeutic efficacy in ameliorating muscle atrophy in myocyte cultures and improving muscle functionality in dexamethasone-treated mice [29]. The convergence of these documented therapeutic properties with the KLP-KM2-mediated alterations in gut microbiota composition observed in our human trial suggests significant potential for KLP-KM2 as a safe postbiotic intervention in the management of sarcopenia, characterized by age-associated decline in muscle mass, strength, and function.

Future investigations would benefit substantially from analyses of the metabolic functional aspects of gut microbiome using the current metagenomic shotgun sequencing data. While our current study establishes the safety profile and taxonomic alterations induced by KLP-KM2, functional analyses would further elucidate the mechanistic underpinnings of these changes by providing detailed insights into the metabolic pathways within the microbial community which are potentially relevant to KLP-KM2's benefits. This comprehensive functional characterization also would bridge the gap between observed taxonomic changes and physiological outcomes, potentially identifying key metabolic pathways and molecular mechanisms that mediate KLP-KM2's beneficial effects on host health, thereby advancing our understanding beyond compositional changes to functional implications in the context of muscle health and aging.

## Supplemental Materials

Supplementary data for this paper are available on-line only at http://jmb.or.kr.



## Figures and Tables

**Table 1:** Chemical composition of study drugs.

	KLP-KM2	Placebo
Calorie (Kcal/100g)	326.62	303.66
Carbohydrate (%)	20.75	88.81
Crude protein (%)	47.21	2.16
Crude lipid (%)	9.88	0.64
Moisture (%)	12.45	7.34
Ash (%)	9.71	1.05
Sodium (mg/100g)	1283.64	160.44
Dietary fiber (%)	17.07	32.99
Crude fiber (%)	3.28	10.60

## References

[1] Goossens D, Jonkers D, Russel M, Stobberingh E, Van Den Bogaard A, StockbrUgger R (2003). The effect of *Lactobacillus plantarum* 299v on the bacterial composition and metabolic activity in faeces of healthy volunteers: a placebo-controlled study on the onset and duration of effects. Aliment. Pharmacol. Ther..

[2] Fidanza M, Panigrahi P, Kollmann TR (2021). *Lactiplantibacillus plantarum*- nomad and ideal probiotic. Front. Microbiol..

[3] Devi MB, Bhattacharya A, Kumar A, Singh CT, Das S, Sarma HK (2024). Potential probiotic *Lactiplantibacillus plantarum* strains alleviate TNF-α by regulating ADAM17 protein and ameliorate gut integrity through tight junction protein expression in in vitro model. Cell Commun. Signal..

[4] Martoni CJ, Srivastava S, Damholt A, Leyer GJ (2023). Efficacy and dose response of *Lactiplantibacillus plantarum* in diarrheapredominant irritable bowel syndrome. World J. Gastroenterol..

[5] Zhang K, Liu H, Liu P, Feng Q, Gan L, Yao L (2023). Positive efficacy of *Lactiplantibacillus plantarum* MH-301 as a postoperative adjunct to endoscopic sclerotherapy for internal hemorrhoids: a randomized, double-blind, placebo-controlled trial. Food Funct..

[6] Elhalik MA, Mekky AE, Khedr M, Suleiman WB (2024). Antineoplastic with DNA fragmentation assay and anti-oxidant, antiinflammatory with gene expression activity of *Lactobacillus plantarum* isolated from local Egyptian milk products. BMC Microbiol..

[7] Zaghloul EH, Ibrahim MIA (2022). Production and characterization of exopolysaccharide from newly isolated marine probiotic *Lactiplantibacillus plantarum* EI6 with in vitro wound healing activity. Front. Microbiol..

[8] Rocchetti MT, Russo P, Capozzi V, Drider D, Spano G, Fiocco D (2021). Bioprospecting antimicrobials from *Lactiplantibacillus plantarum*: key factors underlying its probiotic action. Int. J. Mol. Sci..

[9] Borchers AT, Selmi C, Meyers FJ, Keen CL, Gershwin ME (2009). Probiotics and immunity. J. Gastroenterol..

[10] Adams M, Nout Fermentation and Food Safety.

[11] Lee M-G, Kang MJ, Cha S, Kim T-R, Park Y-S (2024). Acid tolerance responses and their mechanisms in *Lactiplantibacillus plantarum* LM1001. Food Sci. Biotechnol..

[12] Kleerebezem M, Boekhorst J, van Kranenburg R, Molenaar D, Kuipers OP, Leer R (2003). Complete genome sequence of *Lactobacillus plantarum* WCFS1. Proc. Natl. Acad. Sci. USA.

[13] Ricci A, Allende A, Bolton D, Chemaly M, Davies R, EFSA Panel on Biological Hazards (BIOHAZ) (2017). Update of the list of QPS-recommended biological agents intentionally added to food or feed as notified to EFSA 5: suitability of taxonomic units notified to EFSA until September 2016. EFSA J..

[14] Zawistowska-Rojek A, Tyski S (2018). Are probiotic really safe for humans?. Pol. J. Microbiol..

[15] Kullar R, Goldstein EJC, Johnson S, McFarland LV (2023). *Lactobacillus* bacteremia and probiotics: a review. Microorganisms.

[16] Doron S, Snydman DR (2015). Risk and safety of probiotics. Clin. Infect. Dis..

[17] Vinderola G, Sanders ME, Salminen S (2022). The concept of postbiotics. Foods.

[18] Ma L, Tu H, Chen T (2023). Postbiotics in human health: a narrative review. Nutrients.

[19] Aguilar-Toalá JE, Garcia-Varela R, Garcia HS, Mata-Haro V, González-Córdova AF, Vallejo-Cordoba B (2018). Postbiotics: an evolving term within the functional foods field. Trends Food Sci. Technol..

[20] Żółkiewicz J, Marzec A, Ruszczyński M, Feleszko W (2020). Postbiotics-A step beyond pre- and probiotics. Nutrients.

[21] Vinderola G, Sanders ME, Salminen S, Szajewska H (2022). Postbiotics: the concept and their use in healthy populations. Front. Nutr..

[22] Kim JH, Kwak W, Nam Y, Baek J, Lee Y, Yoon S (2024). Effect of postbiotic *Lactiplantibacillus plantarum* LRCC5314 supplemented in powdered milk on type 2 diabetes in mice. J. Dairy Sci..

[23] Guan L, Hu A, Ma S, Liu J, Yao X, Ye T (2024). *Lactiplantibacillus plantarum* postbiotic protects against *Salmonella* infection in broilers via modulating NLRP3 inflammasome and gut microbiota. Poult. Sci..

[24] Li Y, Zhen S, Cao L, Sun F, Wang L (2023). Effects of *Lactobacillus plantarum* postbiotics on growth performance, immune status, and intestinal microflora of growing minks. Animals.

[25] Hu A, Huang W, Shu X, Ma S, Yang C, Zhang R (2023). *Lactiplantibacillus plantarum* postbiotics suppress *Salmonella* infection via modulating bacterial pathogenicity, autophagy and inflammasome in mice. Animals.

[26] Lee J, Park S, Oh N, Park J, Kwon M, Seo J (2021). Oral intake of *Lactobacillus plantarum* L-14 extract alleviates TLR2- and AMPK-mediated obesity-associated disorders in high-fat-diet-induced obese C57BL/6J mice. Cell Prolif..

[27] Heo S, Kim JH, Kwak MS, Jeong DW, Sung MH (2021). Complete genome sequence of *Lactiplantibacillus plantarum* KM2 from lowtemperature aging beef. Korean J. Microbiol..

[28] Heo S, Jung EJ, Park MK, Sung MH, Jeong DW (2024). Evolution and competitive struggles of *Lactiplantibacillus plantarum* under different oxygen contents. Int. J. Mol. Sci..

[29] Jeong YJ, Kim JH, Jung YJ, Kwak MS, Sung MH, Imm JY (2024). KL-Biome (Postbiotic Formulation of *Lactiplantibacillus plantarum* KM2) Improves dexamethasone-induced muscle atrophy in mice. Int. J. Mol. Sci..

[30] Bolger AM, Lohse M, Usadel B (2014). Trimmomatic: a flexible trimmer for Illumina sequence data. Bioinformatics.

[31] Li H (2013). Aligning sequence reads, clone sequences and assembly contigs with BWA-MEM.

[32] Li H, Handsaker B, Wysoker A, Fennell T, Ruan J, Homer N (2009). The sequence alignment/map format and SAMtools. Bioinformatics.

[33] Wood DE, Salzberg SL (2014). Kraken: ultrafast metagenomic sequence classification using exact alignments. Genome Biol..

[34] Lu J, Breitwieser FP, Thielen P, Salzberg SL (2017). Bracken: estimating species abundance in metagenomics data. PeerJ. Comput. Sci..

[35] Oksanen J, Blanchet FG, Friendly M, Kindt R, Legendre P, McGlinn D (2019). vegan: community ecology package.

[36] Paradis E, Claude J, Strimmer K (2004). APE: analyses of phylogenetics and evolution in R language. Bioinformatics.

[37] Wickham H (2016). ggplot2: elegant graphics for data analysis.

[38] Mallick H, Rahnavard A, McIver LJ, Ma S, Zhang Y, Nguyen LH (2021). Multivariable association discovery in population-scale meta-omics studies. PLoS Comput. Biol..

[39] Zhang SM, Huang SL (2023). The commensal anaerobe *Veillonella dispar* reprograms its lactate metabolism and short-chain fatty acid production during the stationary phase. Microbiol. Spectr..

[40] Han M, Liu G, Chen Y, Wang D, Zhang Y (2020). Comparative genomics uncovers the genetic diversity and characters of *Veillonella atypica* and provides insights into its potential applications. Front. Microbiol..

[41] Scheiman J, Luber JM, Chavkin TA, MacDonald T, Tung A, Pham L-D (2019). Meta-omics analysis of elite athletes identifies a performance-enhancing microbe that functions via lactate metabolism. Nat. Med..

[42] Wicaksono DP, Washio J, Abiko Y, Domon H, Takahashi N (2020). Nitrite production from nitrate and its link with lactate metabolism in oral *Veillonella* spp. Appl. Environ. Microbiol..

[43] Kolenbrander P, Dworkin M, Falkow S, Rosenberg E, Schleifer K-H, Stackebrandt E (2006). The Prokaryotes: Volume 4: Bacteria: *Firmicutes*, Cyanobacteria.

[44] Ng SK, Hamilton IR (1973). Carbon dioxide fixation by *Veillonella parvula* M4 and its relation to propionic acid formation. Can. J. Microbiol..

[45] Yutin N, Galperin MY (2013). A genomic update on clostridial phylogeny: Gram-negative spore formers and other misplaced clostridia. Environ. Microbiol..

[46] Rey FE, Faith JJ, Bain J, Muehlbauer MJ, Stevens RD, Newgard CB (2010). Dissecting the in vivo metabolic potential of two human gut acetogens. J. Biol. Chem..

[47] Parker BJ, Wearsch PA, Veloo ACM, Rodriguez-Palacios A (2020). The genus Alistipes: gut bacteria with emerging implications to inflammation, cancer, and mental health. Front. Immunol..

[48] Zhu L, Suhr Van Haute MJ, Hassenstab HR, Smith C, Rose DJ, Mustoe AC (2020). Fecal short-chain fatty acid concentrations increase in newly paired male marmosets (*Callithrix jacchus*). mSphere.

[49] Franko B, Fournier P, Jouve T, Malvezzi P, Pelloux I, Brion JP (2017). *Lactobacillus* bacteremia: pathogen or prognostic marker?. Med. Mal. Infect..

[50] Cannon JP, Lee TA, Bolanos JT, Danziger LH (2005). Pathogenic relevance of *Lactobacillus*: a retrospective review of over 200 cases. Eur. J. Clin. Microbiol. Infect. Dis..

[51] Nikolich-Žugich J (2018). The twilight of immunity: emerging concepts in aging of the immune system. Nat. Immunol..

[52] Pera A, Campos C, López N, Hassouneh F, Alonso C, Tarazona R (2015). Immunosenescence: implications for response to infection and vaccination in older people. Maturitas.

[53] Aw D, Silva AB, Palmer DB (2007). Immunosenescence: emerging challenges for an ageing population. Immunology.

[54] Wilkins T, Sequoia J (2017). Probiotics for gastrointestinal conditions: a summary of the evidence. Am. Fam. Phys..

[55] Bafeta A, Koh M, Riveros C, Ravaud P (2018). Harms reporting in randomized controlled trials of interventions aimed at modifying microbiota: a systematic review. Ann. Intern. Med..

[56] Sanders ME, Lenoir-Wijnkoop I, Salminen S, Merenstein DJ, Gibson GR, Petschow BW (2014). Probiotics and prebiotics: prospects for public health and nutritional recommendations. Ann. NY Acad. Sci..

