# Supplementary material for: Evaluation of the Safety and Impact of Heat-Treated Lactiplantibacillus plantarum KM2 Fermentation on Gut Microbiome Architecture
Source: J Microbiol Biotechnol. 2024 Dec 27;35:e2411069. doi: 10.4014/jmb.2411.11069 (PMC11813362; doi:10.4014/jmb.2411.11069)

<u>Supplementary Information</u> for:

"Evaluation of the safety and impact of heat-treated *Lactiplantibacillus plantarum* KM2 fermentation on gut microbiome architecture"

Seok Won [a, 1], Yejin Jeong [b, 1], Ji-Eun Kim [b], Jong-Hun Kim [b], Hyun Soo Song [a], Hyun-Hwa Bae [c], Mi-Sun Kwak [b], Don-Kyu Kim [c,*], Moon-Hee Sung [b,*], Suryang Kwak [a,*]

[a] *Department of Bio and Fermentation Convergence Technology, College of Science and Technology, Kookmin University, Seoul 02707, Republic of Korea*

[b] *Microbiome Technology Research Institute, Kookmin Bio, Seoul 02826, Republic of Korea*

[c] *Department of Physical Medicine and Rehabilitation, Chung-Ang University Gwangmyeong Hospital, Gwangmyeong 14353, Republic of Korea*

* Corresponding authors

Don-Kyu Kim (+82-2-2610-9003, donkim21@cau.ac.kr)

Moon-Hee Sung (+82-2-927-2024, smoonhee@kmbio.co.kr)

Suryang Kwak (+82-2-910-5735, skwak@kookmin.ac.kr)

Design of the randomized, double-blind, placebo-controlled human study

To maintain the double-blinding, in addition to the information mentioned in the labeling and packaging of study drugs, the assignment details of the unique codes for each treatment group (information on the blinding) were managed by the principal investigators of this study in a sealed state. Except for cases where it was unavoidably necessary to access the code due to the occurrence of a serious adverse event or other important clinical situations, the codes were not to be disclosed until the completion of the human study. Investigators of this human study provided the subjects with the study drugs matching the randomly assigned number, and in the event of a deficiency or damage to the clinical trial product, used the spare product (with the unique code) to maintain the blinding. The records that could identify the subjects in the clinical trial were strictly kept confidential. All documents related to this human study were identified using participant codes only without actual names. The subjects were informed that all trial data would be handled as strictly confidential information.

Figure S1. Rarefaction analysis of sequencing data of this study based on alpha diversity after taxonomic classification.

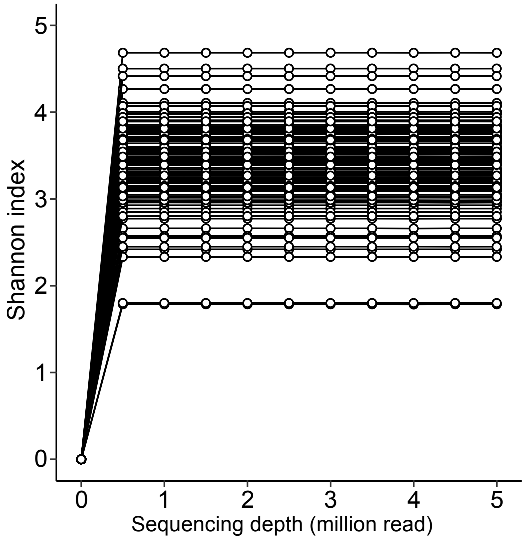

Supplement: Supplementary file 1 [file jmb-35-e2411069-supple.pdf]
